# Supplementary material for: Divergent Immune Pathways in Coronary Artery Disease and Aortic Stenosis: The Role of Chronic Inflammation and Senescence
Source: Int J Mol Sci. 2025 May 29;26(11):5248. doi: 10.3390/ijms26115248 (PMC12154296; doi:10.3390/ijms26115248)
Supplement: Supplementary file 1 [file ijms-26-05248-s001.zip › ijms-3602935-supplementary.pdf]

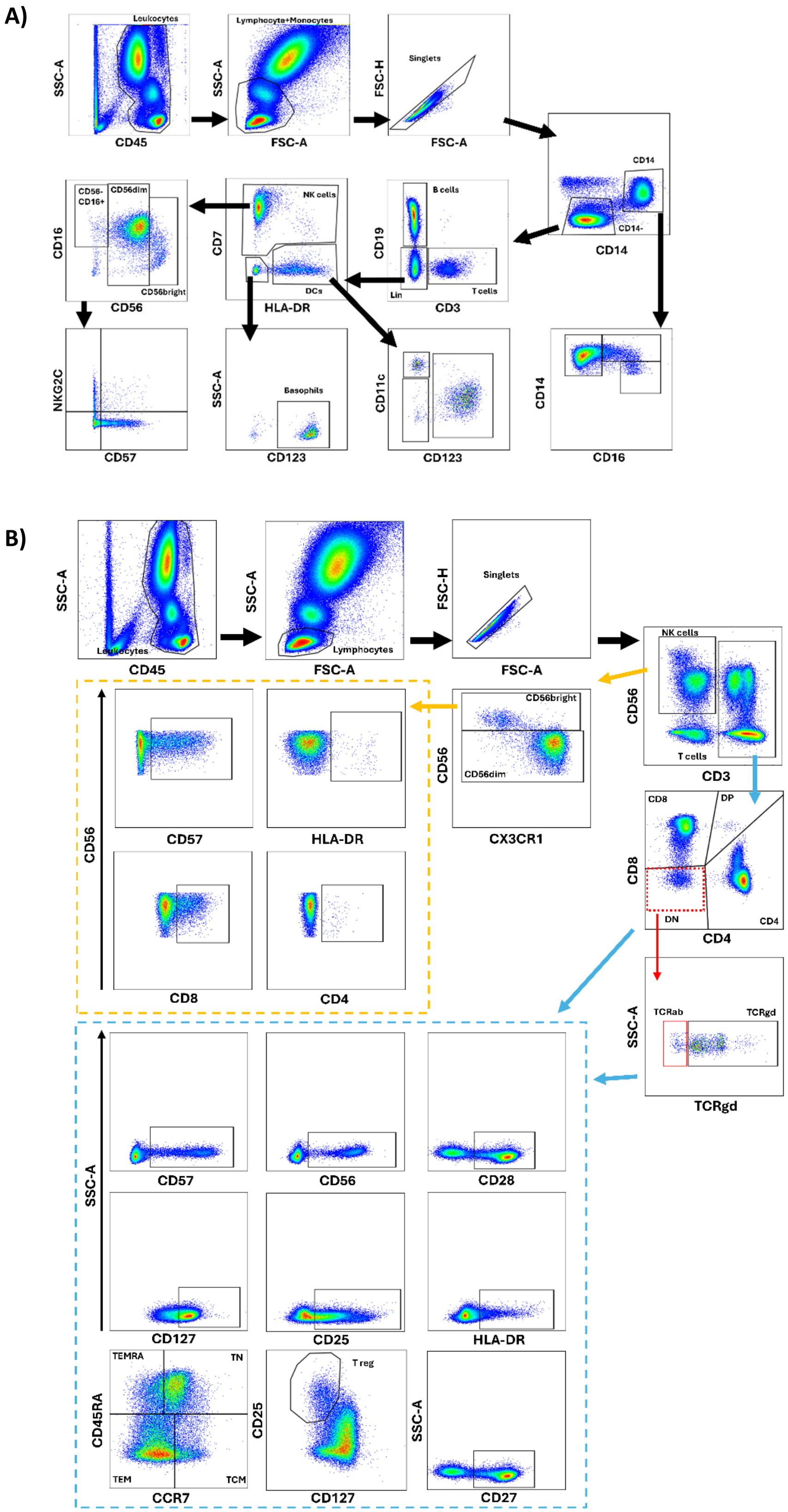

Figure S1. Gating strategy used for A) Innate panel analysis and B) T-cell panel analysis. The figure shows an example of a CAD patient.

Table S1. Study cohort demographics

Parameters	Controls (n = 36)	iCAD (n = 20)	ASCAD (n = 16)	*p*-value
**Basic characteristics**				
Age (years)	66.5	66.8	66.5	0.993
Male, n (%)	28 (77,8)	14 (70)	14 (87,5)	0.455
BMI (kg/m2)	26,3±4,1	28,3±4,3	29,5±5,6	0.046
Ever smoker, n (%)	18 (50)	13 (65)	8 (50)	0.519
Hypertension, n (%)	15 (41,6)	17 (85)	13 (81,3)	0.001
Dyslipidemia, n (%)	7 (19,4)	16 (80)	12 (75)	0
Diabetes, n (%)	1 (2,8)	11 (55)	6 (37,5)	0
**Laboratory examinations**				
Leukocytes (x10^3/mL)	7,1±2	7,8±2	8,3±2,2	0.125
Neutrophils (x10^3/mL)	4,2±1,4	4,7±1,7	5,8±1,9	0.007
Monocytes (x10^3/mL)	0,6±0,2	0,5±0,2	0,5±0,1	0.166
Lymphocytes (x10^3/mL)	2,2±0,7	2±0,6	1,7±0,6	0.053

Table S2. Flow cytometry antibody staining panel (T cell panel).

Antibody	Fluorochrome	Clone	Supplier	Cat. No.
CD45	Alexa Fluor 700	HI30	BD Beckton Dickison	560566
CD3	PE Cy 5.5	SK7	Invitrogen	35-0036-42
CD8	APC-H7	SK1	BD Beckton Dickison	562423
CD4	BB700	SK3	BD Beckton Dickison	566392
CD28	PE Cy 7	CD28.2	BD Beckton Dickison	560684
CD56	BV421	NCAM16.2	BD Beckton Dickison	562751
CD27	APC	M-T271	BD Beckton Dickison	558664
CD57	FITC	NK-1	BD Beckton Dickison	555619
CCR7	BV786	3D12	Invitrogen	78-1977-41
CD45RA	BV605	HI100	BD Beckton Dickison	562886
CD127	BV711	HIL-7R-M21	BD Beckton Dickison	563165
CD25	PE	M-A251	BD Beckton Dickison	555432
HLA-DR	BV570	L243	Biolegend	307638
CX3CR1	PE CF594	2A9-1	BD Beckton Dickison	565897
TCRγδ	BV650	B1	BD Beckton Dickison	564156

Table S3. Flow cytometry antibody staining panel (Innate cells panel).

Antibody	Flurocrome	Clone	Supplier	Cat. No
CD45	Alexa Fluor 700	HI30	BD Beckton Dickison	560566
CD3	PE Cy 5.5	SK7	Invitrogen	35-0036-42
CD56	BV421	NCAM16.2	BD Beckton Dickison	562751
CD14	APC-Vio-770	TÜK4	Miltenyi	130-113-706
CD19	BV650	SJ25C1	BD Beckton Dickison	563226
CD16	BV786	3G8	BD Beckton Dickison	563690
CD7	APC	M-T701	BD Beckton Dickison	561604
CD57	FITC	NK-1	BD Beckton Dickison	555619
NKG2C	PE	REA205	Miltenyi	130-119-776
CD123	PE Cy 7	7G3	BD Beckton Dickison	560826
HLA-DR	BV570	L243	Biolegend	307638
CD11c	PE CF594	3.9	BD Beckton Dickison	565920

Table S4. Absolute counts and percentages of NK cell subsets among study groups.

	ABSOLUTE NUMBERS								PERCENTAGES							
	mean				p-value				mean				p-value			
	HD	CAD	iCAD	ASCAD	HD vs CAD	HD vs iCAD	HD vs ASCAD	iCAD vs ASCAD	HD	CAD	iCAD	ASCAD	HD vs CAD	HD vs iCAD	HD vs ASCAD	iCAD vs ASCAD
CD56bright	0,0119	0,0138	0,0135	0,0143	0,2310	0,1222	0,7555	0,3859	3,3116	6,1407	7,4955	4,4472	**0,0001**	**0,0000**	0,0984	**0,0196**
CD56bright CD57+NKG2C+	0,0004	0,0004	0,0005	0,0003	*0,0504*	**0,0382**	0,2929	0,2018	2,3236	3,4931	3,7143	3,2166	*0,0561*	**0,0434**	0,3025	0,4630
CD56bright CD57+NKG2C-	0,0005	0,0014	0,0020	0,0006	**0,0002**	**0,0000**	0,1898	**0,0001**	4,2560	11,6800	17,2434	4,7257	**0,0001**	**0,0000**	0,1300	**0,0001**
CD56bright CD57-NKG2C+	0,0020	0,0024	0,0021	0,0027	0,1658	0,4059	0,1411	0,2758	17,4947	17,9193	14,7900	21,8310	0,6496	0,4785	0,1027	**0,0286**
CD56bright CD57-NKG2C-	0,0091	0,0097	0,0089	0,0106	0,6933	0,6088	0,9280	0,5819	75,9257	66,9076	64,2523	70,2267	**0,0030**	**0,0012**	0,1368	0,1480
CD56br NKG2C	0,0023	0,0028	0,0026	0,0030	0,1538	0,3186	0,1770	0,5035	19,8183	21,4124	18,5043	25,0476	0,5781	0,6810	0,1743	0,1104
CD56br CD4	0,0001	0,0001	0,0001	0,0002	0,4769	0,6252	0,4986	0,9619	0,7341	1,3854	0,9521	1,9270	0,1511	0,2911	0,1959	0,5125
CD56br CD8	0,0038	0,0028	0,0025	0,0030	**0,0019**	**0,0058**	**0,0244**	0,7893	26,8761	22,3505	22,5209	22,1375	0,0637	0,1811	0,0902	0,7176
CD56br CX3CR1	0,0030	0,0029	0,0027	0,0031	0,3276	0,3201	0,5877	0,9345	24,8720	23,4871	23,1300	23,9634	0,6067	0,5896	0,8099	0,8474
CD56br HLADR	0,0003	0,0004	0,0005	0,0002	0,2720	**0,0018**	0,0737	**0,0000**	1,8558	3,7308	4,8817	2,1963	**0,0023**	**0,0000**	0,6418	**0,0003**
CD56dim	0,3982	0,2411	0,2014	0,2906	**0,0000**	**0,0000**	**0,0118**	**0,0422**	92,0907	87,2347	87,0842	87,4229	**0,0019**	**0,0025**	*0,0519*	0,8944
CD56dim CD57	0,1805	0,1553	0,1193	0,2003	0,4002	0,1451	0,8487	0,0827	43,8649	58,0329	51,6870	65,9653	**0,0139**	0,2543	**0,0023**	**0,0215**
CD56dim CD57+NKG2C+	0,0372	0,0167	0,0137	0,0204	0,0755	**0,0248**	0,5943	0,1159	9,3530	6,9298	5,2827	8,9887	0,6765	0,4605	0,9121	0,3859
CD56dim CD57+NKG2C-	0,1430	0,1208	0,0782	0,1741	0,4444	0,0672	0,4505	**0,0329**	34,6212	47,7090	41,6647	55,2642	**0,0083**	0,2284	**0,0007**	**0,0392**
CD56dim CD57-NKG2C+	0,0197	0,0084	0,0085	0,0082	**0,0026**	**0,0177**	**0,0111**	0,7652	5,4183	3,8150	4,2255	3,3020	0,2010	0,5151	0,1368	0,5819
CD56dim CD57-NKG2C-	0,1983	0,0952	0,1010	0,0879	**0,0000**	**0,0007**	**0,0004**	0,8875	50,6075	41,5462	48,8271	32,4451	0,0711	0,7557	**0,0016**	**0,0072**
CD56dim NKG2C	0,0569	0,0251	0,0223	0,0286	**0,0070**	**0,0058**	0,1213	0,1681	14,7713	10,7448	9,5082	12,2906	0,1740	0,1882	0,3922	0,6483
CD56dim CD4	0,0025	0,0054	0,0039	0,0072	0,1327	0,2151	0,2303	0,5187	0,8960	2,4726	2,1552	2,8693	**0,0086**	**0,0087**	0,1157	0,8875
CD56dim CD8	0,1263	0,0789	0,0650	0,0963	**0,0042**	**0,0006**	0,2743	*0,0533*	37,4725	34,9215	32,2383	38,2756	0,4022	0,1231	0,8529	0,2008
CD56dim CX3CR1	0,2945	0,2005	0,1779	0,2307	**0,0008**	**0,0013**	**0,0330**	0,2270	90,1229	80,4601	75,7045	86,8008	0,1530	0,1448	0,4303	0,7051
CD56dim HLADR	0,0037	0,0040	0,0051	0,0025	0,7971	0,3119	0,0882	**0,0116**	1,2813	2,0192	2,7222	1,0820	0,1115	**0,0032**	0,5324	**0,0049**
CD56-CD16++	0,0092	0,0114	0,0068	0,0172	0,1497	**0,0028**	0,4035	0,1783	2,4257	4,4714	3,1432	6,1317	0,1891	0,3994	0,1856	0,4402
CD56-CD16++ CD57	0,0033	0,0061	0,0023	0,0108	0,8836	0,1323	0,1396	0,0949	27,6195	30,3112	22,1118	40,5605	0,7257	0,3961	0,1066	**0,0215**

Table S5. Absolute counts and percentages of CD4+ T cell subsets among study groups.

	ABSOLUTE NUMBERS								PERCENTAGES							
	mean				p-value				mean				p-value			
	HD	CAD	iCAD	ASCAD	HD vs CAD	HD vs iCAD	HD vs ASCAD	iCAD vs ASCAD	HD	CAD	iCAD	ASCAD	HD vs CAD	HD vs iCAD	HD vs ASCAD	iCAD vs ASCAD
CD4 CD25	0,7221	0,3333	0,3515	0,3106	**0,0000**	**0,0000**	**0,0000**	0,4919	47,1060	43,8764	42,9344	45,0539	0,2414	0,1435	0,6252	0,6216
CD4 CD127	0,5946	0,4613	0,5087	0,4020	0,1363	0,3370	0,1355	0,2234	65,3561	60,9073	60,5311	61,3776	**0,0290**	**0,0104**	0,2222	0,7828
CD4 CD56	0,4061	0,0235	0,0213	0,0262	**0,0056**	**0,0457**	**0,0111**	0,3688	1,9723	3,2906	2,9037	3,7742	0,3728	0,3818	0,5893	0,8875
CD4 CX3CR1	0,0806	0,0492	0,0436	0,0565	**0,0468**	0,0710	0,1711	0,9084	6,2405	6,7582	5,2921	8,7131	0,9499	0,6169	0,6307	0,4785
CD4 HLADR	0,3017	0,0714	0,0974	0,0368	**0,0015**	0,3725	**0,0000**	**0,0000**	5,5151	9,2520	11,1280	6,7508	**0,0000**	**0,0000**	0,4183	**0,0024**
CD4 CD27	0,8558	0,5820	0,6617	0,4824	**0,0018**	*0,0539*	**0,0011**	0,0667	77,1151	75,2646	77,9117	71,9556	0,9866	0,8322	0,8368	0,5393
CD4 CD28	1,1532	0,6974	0,7879	0,5843	**0,0000**	**0,0005**	**0,0000**	0,0771	94,4033	91,8136	94,2664	88,7476	0,1907	0,4813	0,1409	0,4402
CD4 CD28null	0,1332	0,0572	0,0492	0,0672	0,0687	0,1143	0,1763	0,9124	5,5967	8,1864	5,7337	11,2524	0,1907	0,4813	0,1409	0,4402
CD4 CD57	0,2173	0,0616	0,0447	0,0828	*0,0574*	**0,0386**	0,3500	0,1016	5,7202	8,6094	5,2519	12,8063	0,0741	0,8190	**0,0034**	**0,0028**
CD4 CD28+CD57+	0,0200	0,0180	0,0092	0,0291	0,5319	**0,0333**	0,1744	**0,0044**	1,5444	2,4343	1,1336	4,1685	0,1142	0,6169	**0,0003**	**0,0013**
CD4 CD28+CD57-	1,1945	0,6794	0,7787	0,5552	**0,0000**	**0,0000**	**0,0000**	**0,0422**	92,8590	89,3279	93,1327	84,5719	0,0779	0,7282	**0,0063**	**0,0179**
CD4 CD28-CD57+	0,0534	0,0436	0,0355	0,0538	0,9331	0,9797	0,8522	0,8875	4,1758	6,1238	4,1183	8,6306	0,1618	0,4603	0,1111	0,3859
CD4 CD28-CD57-	0,0186	0,0136	0,0137	0,0134	0,1795	0,4500	0,1409	0,5819	1,4208	2,0627	1,6153	2,6219	0,4101	0,6289	0,3815	0,8381
CD4 CD28-CD57+CX3CR1+	0,0408	0,0270	0,0189	0,0378	0,3812	0,2046	0,9755	0,3640	3,2305	3,7325	2,2801	5,6689	0,6598	0,6779	0,1915	0,1794
CD4 CD28-CD56+CD57+CX3CR1+	0,0070	0,0098	0,0057	0,0152	0,6165	0,4615	1,0000	0,5482	0,5668	1,3162	0,6984	2,1400	0,8045	0,7064	0,3627	0,3589
CD4 CCR7	0,6078	0,4164	0,4180	0,4143	0,0653	0,1224	0,1520	0,9575	68,0656	55,4265	48,7506	63,7713	**0,0000**	**0,0000**	0,2082	**0,0002**
CD4 CD45RA	0,3942	0,3361	0,4023	0,2532	0,6746	0,6289	0,1894	**0,0255**	41,7781	42,6210	46,4312	37,8583	0,8030	0,1794	0,4559	0,1270
CD4 TN	0,4722	0,2426	0,2719	0,2060	**0,0000**	**0,0002**	**0,0000**	0,2099	36,0457	30,8604	30,8933	30,8191	**0,0313**	0,0607	0,1111	0,9880
CD4 TN CD25	0,1516	0,0679	0,0588	0,0792	**0,0000**	**0,0000**	**0,0024**	0,9124	30,6292	25,9180	22,0520	30,7505	**0,0233**	**0,0022**	0,9813	0,1317
CD4 TN CD27	0,4592	0,2356	0,2674	0,1958	**0,0000**	**0,0003**	**0,0000**	0,1782	96,5993	93,1014	97,2286	87,9425	0,4295	0,0796	0,5361	0,1999
CD4 TN CD28	0,4636	0,2371	0,2693	0,1968	**0,0000**	**0,0003**	**0,0000**	0,1739	97,6778	94,3019	98,7723	88,7139	0,5342	0,3635	**0,0289**	**0,0112**
CD4 TN CD56	0,0058	0,0039	0,0022	0,0060	**0,0045**	**0,0040**	0,0981	0,9875	1,5022	2,7567	1,4561	4,3825	0,9420	0,3201	0,3107	0,1681
CD4 TN CD57	0,0085	0,0053	0,0026	0,0087	0,1363	**0,0113**	0,7911	**0,0101**	2,1617	4,2668	1,1525	8,1597	0,6104	0,2508	**0,0233**	**0,0028**

CD4 TN CD127	0,3284	0,1652	0,1888	0,1357	**0,0000**	**0,0009**	**0,0000**	0,1317	69,6063	65,8382	68,5428	62,4574	0,1518	0,5358	0,0725	0,1504
CD4 TN CX3CR1	0,0130	0,0058	0,0036	0,0086	**0,0006**	**0,0002**	0,1007	*0,0503*	3,2729	4,2765	1,3574	8,1687	0,1814	**0,0097**	0,5058	**0,0173**
CD4 TN HLADR	0,0108	0,0198	0,0265	0,0109	0,3219	**0,0119**	0,2293	**0,0129**	2,6053	8,2536	9,2572	6,9154	**0,0000**	**0,0000**	**0,0060**	0,1794
CD4 TM	0,8171	0,5120	0,5651	0,4455	**0,0000**	**0,0003**	**0,0000**	0,1271	64,1196	69,1397	69,1067	69,1809	0,1003	0,1168	0,2918	0,9880
CD4 TM CD25	0,4608	0,2655	0,2928	0,2314	**0,0000**	**0,0000**	**0,0000**	0,1726	56,9210	51,9015	52,4462	51,2206	*0,0530*	0,0710	0,1962	0,7927
CD4 TM CD27	0,5399	0,3464	0,3942	0,2867	**0,0000**	**0,0033**	**0,0001**	0,0820	66,3727	67,0979	69,8235	63,6908	0,7958	0,2468	0,5482	0,2114
CD4 TM CD28	0,7508	0,4603	0,5186	0,3875	**0,0000**	**0,0002**	**0,0000**	0,0760	92,4485	89,8403	92,1916	86,9012	0,2500	0,4813	0,2330	0,4984
CD4 TM CD56	0,0185	0,0196	0,0191	0,0202	0,5028	0,9251	0,1941	0,2234	2,2895	3,6641	3,5186	3,8459	0,2740	0,1648	0,7610	0,6037
CD4 TM CD57	0,0648	0,0563	0,0421	0,0741	0,9509	0,5246	0,5361	0,1236	7,4695	11,0820	7,1594	15,9852	*0,0589*	0,8322	**0,0017**	**0,0057**
CD4 TM CD127	0,5121	0,2961	0,3199	0,2663	**0,0000**	**0,0000**	**0,0000**	0,2726	62,7139	57,6643	56,4489	59,1836	**0,0149**	**0,0008**	0,3027	0,4076
CD4 TM CX3CR1	0,0658	0,0434	0,0400	0,0479	0,0647	0,1183	0,1584	0,9869	7,1256	8,3777	7,0172	10,1917	0,7360	0,9392	0,6162	0,5644
CD4 TM HLADR	0,0561	0,0516	0,0709	0,0259	*0,0540*	0,4886	**0,0000**	**0,0000**	7,9893	10,0453	12,0278	7,4019	**0,0443**	**0,0004**	0,6162	**0,0024**
CD4 TCM	0,4059	0,1737	0,1461	0,2083	**0,0000**	**0,0000**	**0,0000**	0,1059	32,0200	24,5661	17,8573	32,9522	**0,0001**	**0,0000**	0,6731	**0,0015**
CD4 TCM CD25	0,2456	0,0989	0,0872	0,1135	**0,0000**	**0,0000**	**0,0000**	0,4592	60,3552	58,6781	61,1366	55,6051	0,3550	0,6903	0,2490	0,1889
CD4 TCM CD27	0,3092	0,1344	0,1182	0,1548	**0,0000**	**0,0000**	**0,0000**	0,4592	76,1283	77,6400	80,5742	73,9723	0,0859	**0,0249**	0,8676	0,2487
CD4 TCM CD28	0,3991	0,1681	0,1448	0,1971	**0,0000**	**0,0000**	**0,0000**	0,1643	98,3267	97,1406	99,1025	94,6881	0,5393	0,6410	0,1023	0,0978
CD4 TCM CD56	0,0045	0,0028	0,0011	0,0050	**0,0001**	**0,0000**	**0,0246**	0,6258	1,1623	1,3581	0,8437	2,0011	0,5717	0,4919	0,8522	0,6711
CD4 TCM CD57	0,0118	0,0103	0,0021	0,0204	0,1166	**0,0001**	0,1204	**0,0000**	2,8093	4,6745	1,4300	8,7300	0,2363	0,1143	**0,0000**	**0,0000**
CD4 TCM CD127	0,2794	0,1175	0,1016	0,1374	**0,0000**	**0,0000**	**0,0000**	0,1656	68,6223	67,7819	69,6615	65,4325	0,7582	0,4907	0,2514	0,1129
CD4 TCM CX3CR1	0,0134	0,0070	0,0032	0,0120	**0,0005**	**0,0002**	0,0881	**0,0463**	3,4334	3,6413	2,5598	5,0833	0,5636	0,2879	0,8141	0,2401
CD4 TCM HLADR	0,0254	0,0147	0,0167	0,0119	**0,0000**	**0,0038**	**0,0001**	*0,0503*	6,4697	9,6034	11,3872	7,2250	**0,0027**	**0,0000**	0,8943	**0,0191**
CD4 TEM	0,3348	0,2448	0,2886	0,1900	**0,0151**	0,2958	**0,0019**	*0,0533*	26,2019	32,8128	35,7115	29,1895	**0,0060**	**0,0006**	0,3438	*0,0592*
CD4 TEM CD25	0,1870	0,1396	0,1708	0,1005	**0,0271**	0,5584	**0,0011**	**0,0041**	58,8022	56,9776	60,2735	52,8578	0,6190	0,6824	0,2869	0,1826
CD4 TEM CD27	0,1799	0,1420	0,1736	0,1024	0,0902	0,8989	**0,0040**	**0,0083**	55,1615	58,1851	61,1209	54,5154	0,2106	*0,0524*	0,7911	0,5604
CD4 TEM CD28	0,2954	0,2169	0,2635	0,1586	**0,0055**	0,3083	**0,0005**	**0,0135**	90,3941	88,8234	91,8570	85,0313	0,4979	0,8588	0,3300	0,5393
CD4 TEM CD56	0,0086	0,0082	0,0079	0,0085	0,2454	0,9527	**0,0478**	**0,0457**	2,3731	2,9015	2,6161	3,2583	0,9955	0,6903	0,6306	0,3049
CD4 TEM CD57	0,0399	0,0307	0,0240	0,0392	0,6664	0,9662	0,4146	0,3202	9,9257	12,6104	7,9277	18,4638	0,1114	1,0000	**0,0042**	**0,0022**
CD4 TEM CD127	0,1935	0,1373	0,1618	0,1067	**0,0038**	0,1432	**0,0004**	**0,0234**	58,2194	55,7996	55,8705	55,7110	0,2948	0,2369	0,5206	0,9659
CD4 TEM CX3CR1	0,0385	0,0252	0,0264	0,0237	0,2051	0,4299	0,1915	0,5869	9,9957	9,8590	8,5228	11,6406	0,9317	0,7410	0,8141	0,4993

CD4 TEM HLADR	0,0287	0,0293	0,0421	0,0123	0,2093	0,0738	**0,0000**	**0,0000**	9,4584	11,9729	14,2437	8,9451	**0,0384**	**0,0005**	0,7205	**0,0064**
CD4 TEMRA	0,0735	0,0934	0,1304	0,0472	0,3492	**0,0004**	**0,0209**	**0,0000**	5,7325	11,7607	15,5379	7,0392	**0,0000**	**0,0000**	0,1355	**0,0000**
CD4 TEMRA CD25	0,0282	0,0270	0,0348	0,0173	0,9153	0,0766	**0,0233**	**0,0019**	38,8053	30,7132	27,1704	35,1417	**0,0101**	**0,0002**	0,5107	0,1347
CD4 TEMRA CD27	0,0509	0,0700	0,1024	0,0295	0,3155	**0,0003**	**0,0209**	**0,0000**	71,3991	73,5000	78,9198	66,7251	0,4037	0,2302	0,9453	0,3859
CD4 TEMRA CD28	0,0563	0,0754	0,1103	0,0318	0,3912	**0,0001**	**0,0055**	**0,0000**	80,0365	79,1903	85,7809	70,9521	0,8447	0,7029	0,4260	0,2620
CD4 TEMRA CD56	0,0054	0,0086	0,0101	0,0067	0,9506	0,3009	0,2799	0,1016	6,4756	8,8955	7,7114	10,3757	0,9866	0,9527	0,9765	0,9374
CD4 TEMRA CD57	0,0132	0,0153	0,0160	0,0145	0,5948	0,5699	0,7911	0,6258	16,1258	17,1671	11,2191	24,6021	0,6502	0,6169	0,1638	0,1317
CD4 TEMRA CD127	0,0392	0,0413	0,0565	0,0222	0,9865	**0,0256**	**0,0086**	**0,0001**	53,5742	45,3951	42,5627	48,9355	**0,0179**	**0,0028**	0,3403	0,1950
CD4 TEMRA CX3CR1	0,0139	0,0112	0,0104	0,0122	0,3938	0,6410	0,3403	0,6568	17,2407	12,8727	7,6277	19,8660	0,2590	0,0657	0,8621	0,1585
CD4 TEMRA HLADR	0,0020	0,0077	0,0121	0,0018	**0,0015**	**0,0000**	0,5420	**0,0000**	3,0417	6,8916	8,6038	4,6087	**0,0000**	**0,0000**	0,0806	**0,0004**

Table S6. Absolute counts and percentages of CD8+ T cell subsets among study groups.

	ABSOLUTE NUMBERS								PERCENTAGES							
	mean				p-value				mean				p-value			
	HD	CAD	iCAD	ASCAD	HD vs CAD	HD vs iCAD	HD vs ASCAD	iCAD vs ASCAD	HD	CAD	iCAD	ASCAD	HD vs CAD	HD vs iCAD	HD vs ASCAD	iCAD vs ASCAD
CD8 CD25	0,0910	0,0470	0,0466	0,0475	**0,0112**	**0,0205**	0,0792	0,9875	17,5356	13,3522	11,6804	15,4421	0,4101	0,3370	0,7461	0,5819
CD8 CD127	0,2834	0,1706	0,1779	0,1615	**0,0000**	**0,0007**	**0,0000**	0,1491	53,6699	47,2598	47,6079	46,8245	**0,0306**	*0,0567*	0,1303	0,8657
CD8 CD56	0,1073	0,0864	0,0928	0,0784	0,7076	0,6655	0,2409	0,0949	17,2185	21,8544	24,5832	18,4434	0,0707	**0,0268**	0,5454	0,1402
CD8 CX3CR1	0,2031	0,1455	0,1475	0,1429	*0,0508*	0,2653	**0,0313**	0,2979	33,3776	39,4595	38,4764	40,7704	0,2388	0,4069	0,3553	0,8030
CD8 HLADR	0,0708	0,0713	0,0832	0,0555	0,1281	0,7797	**0,0158**	**0,0427**	14,1004	17,4129	20,0421	13,9074	0,0991	**0,0043**	0,6750	**0,0253**
CD8 CD27	0,2959	0,1586	0,1688	0,1458	**0,0000**	**0,0000**	**0,0000**	0,6711	58,0327	47,0902	45,3991	49,2040	**0,0090**	**0,0055**	0,1697	0,5685
CD8 CD28	0,3487	0,1753	0,1963	0,1490	**0,0000**	**0,0000**	**0,0000**	0,2620	66,9271	51,5534	53,1496	49,5582	**0,0031**	**0,0036**	**0,0192**	0,6245
CD8 CD28null	0,1985	0,2228	0,1792	0,2773	0,6746	0,8588	0,5584	0,8566	33,0729	48,4466	46,8504	50,4418	**0,0031**	**0,0036**	**0,0192**	0,6245
CD8 CD57	0,1574	0,1663	0,1320	0,2092	0,9777	0,7928	0,7166	0,3521	25,6818	38,7439	35,4522	42,8585	**0,0007**	**0,0099**	**0,0067**	0,2206
CD8 CD28+CD57+	0,0297	0,0153	0,0113	0,0204	**0,0056**	**0,0010**	0,2743	0,1783	5,4118	4,9594	3,4248	6,8776	0,3377	*0,0584*	0,6306	0,0667
CD8 CD28+CD57-	0,3190	0,1600	0,1850	0,1286	**0,0000**	**0,0000**	**0,0000**	0,1086	61,5154	46,5940	49,7248	42,6806	**0,0005**	**0,0097**	**0,0066**	0,3041
CD8 CD28-CD57+	0,1277	0,1510	0,1207	0,1888	0,5342	0,3119	0,9453	0,2234	20,2701	33,7845	32,0275	35,9808	**0,0005**	**0,0017**	**0,0132**	0,5327

CD8 CD28-CD57-	0,0708	0,0718	0,0585	0,0884	0,0705	0,3119	**0,0455**	0,0827	12,8028	14,6620	14,8229	14,4609	0,6993	0,4919	0,9141	0,6483
CD8 CD28-CD57+CX3CR1+	0,1088	0,0863	0,0834	0,0901	0,3628	0,6779	0,2631	0,5869	17,0635	22,9902	21,6813	24,7355	0,1852	0,4006	0,1777	0,6155
CD8 CD28-CD56+CD57+CX3CR1+	0,0432	0,0342	0,0377	0,0297	0,5714	0,8855	0,4066	0,5869	6,4059	8,9409	9,6111	8,0472	0,4132	0,4299	0,6162	0,9345
CD8 CCR7	0,0825	0,0560	0,0565	0,0554	**0,0101**	**0,0476**	**0,0274**	0,4592	19,5039	17,1789	15,7838	18,9228	0,5123	0,2302	0,8215	0,1086
CD8 CD45RA	0,3837	0,2839	0,2663	0,3059	**0,0005**	**0,0054**	**0,0014**	**0,0256**	70,1913	67,4966	70,9633	63,1631	0,3286	0,7942	0,1012	0,0956
CD8 TN	0,0179	0,0204	0,0044	0,0404	0,5268	0,4299	**0,0393**	**0,0196**	17,8616	15,6459	14,7302	16,7905	0,5051	0,3457	0,9453	0,3049
CD8 TN CD25	0,0017	0,0015	0,0002	0,0031	**0,0439**	**0,0001**	0,3603	**0,0002**	11,0955	5,6654	2,6874	9,3878	**0,0077**	**0,0002**	0,6875	**0,0031**
CD8 TN CD27	0,0091	0,0097	0,0001	0,0217	0,1584	**0,0000**	**0,0104**	**0,0000**	81,0414	63,3366	61,0793	66,1581	**0,0022**	**0,0006**	0,1520	0,5671
CD8 TN CD28	0,0165	0,0090	0,0027	0,0169	0,1140	**0,0016**	0,4375	**0,0074**	80,2105	56,8539	60,1664	52,7133	**0,0000**	**0,0005**	**0,0001**	0,3987
CD8 TN CD56	0,0033	0,0040	0,0007	0,0082	0,2740	0,8190	**0,0260**	**0,0149**	9,4984	21,1941	24,0092	17,6752	**0,0001**	**0,0004**	**0,0087**	0,4592
CD8 TN CD57	0,0043	0,0073	0,0009	0,0152	**0,0008**	*0,0539*	**0,0002**	**0,0039**	12,0341	26,3979	25,6671	27,3115	**0,0001**	**0,0002**	**0,0104**	0,7957
CD8 TN CD127	0,0086	0,0100	0,0014	0,0208	**0,0361**	0,5815	**0,0019**	**0,0044**	77,1213	72,7187	75,8715	68,7777	0,0968	0,1648	0,1962	0,4216
CD8 TN CX3CR1	0,0073	0,0084	0,0019	0,0164	0,7328	0,0766	0,1520	**0,0359**	20,3256	24,4753	23,1235	26,2776	0,9772	0,7797	0,6901	1,0000
CD8 TN HLADR	0,0009	0,0071	0,0010	0,0149	0,5490	0,3038	**0,0221**	**0,0494**	15,9857	24,0637	22,9000	25,6153	**0,0407**	0,0738	0,1351	0,9084
CD8 TM	0,0739	0,1176	0,0346	0,2213	0,6911	**0,0196**	**0,0478**	**0,0054**	81,8001	84,3541	85,2698	83,2095	0,5051	0,3457	0,9453	0,3049
CD8 TM CD25	0,0654	0,0395	0,0322	0,0485	0,1220	**0,0339**	0,7911	0,3202	19,0927	14,9342	13,2428	17,0486	0,4037	0,3285	0,7461	0,7176
CD8 TM CD27	0,0429	0,0503	0,0159	0,0934	0,5717	**0,0074**	**0,0393**	**0,0060**	54,2415	44,1197	43,0223	45,4913	**0,0146**	**0,0109**	0,1598	0,6940
CD8 TM CD28	0,1113	0,0727	0,0516	0,0989	**0,0351**	**0,0007**	0,9141	0,0620	65,2338	50,9525	52,1943	49,4002	**0,0022**	**0,0066**	**0,0339**	0,7028
CD8 TM CD56	0,0462	0,0352	0,0234	0,0499	0,3492	0,2302	0,8063	0,5187	18,7844	22,0608	24,4756	19,0423	0,1907	0,0857	0,7610	0,2358
CD8 TM CD57	0,0413	0,0726	0,0201	0,1383	0,0670	0,7668	**0,0034**	**0,0060**	28,1491	41,0367	36,8997	46,2079	**0,0011**	**0,0248**	**0,0049**	0,1315
CD8 TM CD127	0,0596	0,0772	0,0306	0,1354	0,7076	0,2508	**0,0433**	**0,0054**	49,1659	42,9629	43,0547	42,8481	**0,0372**	*0,0570*	0,1574	0,9640
CD8 TM CX3CR1	0,0732	0,0633	0,0322	0,1022	0,3434	0,0683	0,6731	**0,0494**	35,9461	42,3147	40,9954	44,0739	0,4399	0,5470	0,5190	0,7456
CD8 TM HLADR	0,0183	0,0225	0,0180	0,0282	0,9065	0,7029	0,5105	0,3859	13,9348	16,0905	19,5594	11,4653	0,2355	**0,0018**	0,1245	**0,0018**
CD8 TCM	0,0024	0,0028	0,0005	0,0057	0,7328	0,1401	**0,0187**	**0,0123**	1,6423	1,5330	1,0536	2,1322	0,6183	0,1183	0,3603	**0,0350**
CD8 TCM CD25	0,0003	0,0011	0,0000	0,0025	0,8799	0,0738	**0,0177**	**0,0135**	43,3621	41,1936	49,2390	31,1368	0,6655	0,2601	0,0640	**0,0079**
CD8 TCM CD27	0,0014	0,0019	0,0000	0,0041	0,9866	*0,0561*	**0,0233**	**0,0163**	77,7796	71,7726	74,5998	68,2386	**0,0090**	0,1776	**0,0049**	*0,0552*
CD8 TCM CD28	0,0017	0,0022	0,0000	0,0049	0,7328	**0,0092**	**0,0167**	**0,0067**	97,4617	90,0759	95,5732	83,2043	**0,0004**	0,0606	**0,0000**	**0,0095**
CD8 TCM CD56	0,0001	0,0001	0,0000	0,0002	0,2624	0,3602	**0,0023**	**0,0003**	4,0152	5,6543	4,0993	7,5979	0,1036	0,2700	0,1150	0,6037
CD8 TCM CD57	0,0002	0,0005	0,0000	0,0012	0,0679	0,5044	**0,0001**	**0,0001**	5,2280	12,0185	4,5975	21,2949	0,0724	0,5437	**0,0001**	**0,0002**

CD8 TCM CD127	0,0007	0,0013	0,0000	0,0030	0,2347	0,2345	**0,0005**	**0,0003**	54,5806	61,5647	63,9488	58,5846	**0,0217**	**0,0138**	0,2641	0,1114
CD8 TCM CX3CR1	0,0003	0,0012	0,0000	0,0026	0,2408	0,4603	**0,0026**	**0,0017**	8,4533	21,0622	14,7754	29,4446	**0,0090**	0,1104	**0,0060**	0,1487
CD8 TCM HLADR	0,0002	0,0003	0,0000	0,0006	0,0687	0,8455	**0,0024**	**0,0039**	14,9035	23,5181	28,0179	17,5183	**0,0014**	**0,0000**	0,4066	**0,0102**
CD8 TEM	0,0037	0,0315	0,0002	0,0707	0,1584	0,8059	**0,0045**	**0,0060**	28,1663	30,9704	27,9830	34,7047	0,2937	0,9495	0,1073	0,1262
CD8 TEM CD25	0,0113	0,0119	0,0067	0,0184	0,9509	0,1596	0,0792	**0,0235**	24,9842	22,8826	24,9524	20,2955	0,9866	0,4707	0,4260	0,1402
CD8 TEM CD27	0,0090	0,0231	0,0044	0,0465	0,6664	0,0766	**0,0040**	**0,0025**	69,7283	59,4256	62,2612	55,8811	**0,0024**	**0,0074**	**0,0274**	0,2858
CD8 TEM CD28	0,0241	0,0295	0,0130	0,0501	0,7076	**0,0074**	**0,0158**	**0,0060**	85,0741	72,5810	78,9326	64,6416	**0,0045**	0,1701	**0,0005**	0,0771
CD8 TEM CD56	0,0036	0,0040	0,0025	0,0059	0,7582	0,3285	0,0902	**0,0457**	8,9209	10,2845	10,3746	10,1719	0,4979	0,2958	0,9922	0,3688
CD8 TEM CD57	0,0021	0,0124	0,0007	0,0270	**0,0233**	0,7155	**0,0000**	**0,0000**	16,8020	29,0980	20,1390	40,2968	**0,0089**	0,3645	**0,0005**	**0,0033**
CD8 TEM CD127	0,0069	0,0140	0,0023	0,0285	0,1652	0,2580	**0,0001**	**0,0000**	46,9461	44,4342	48,2007	39,7261	0,4487	0,7144	0,1297	0,0815
CD8 TEM CX3CR1	0,0079	0,0131	0,0032	0,0255	0,3377	0,4603	**0,0092**	**0,0060**	21,0569	36,3114	30,5340	44,0147	**0,0164**	0,0923	**0,0237**	0,2827
CD8 TEM HLADR	0,0028	0,0030	0,0015	0,0050	0,1486	0,8190	**0,0040**	**0,0049**	17,1446	16,4150	20,9063	10,4266	0,6516	**0,0386**	**0,0006**	**0,0000**
CD8 TEMRA	0,0183	0,0692	0,0089	0,1445	0,6183	0,1143	**0,0055**	**0,0022**	52,3298	51,8507	56,2332	46,3726	0,8816	0,2050	0,2436	0,0641
CD8 TEMRA CD25	0,0214	0,0130	0,0078	0,0196	*0,0530*	**0,0026**	0,9922	**0,0389**	15,3091	10,4787	7,2939	14,4597	0,1455	**0,0225**	0,9609	**0,0457**
CD8 TEMRA CD27	0,0142	0,0202	0,0031	0,0416	0,3048	**0,0006**	**0,0393**	**0,0017**	45,9450	37,8542	35,0924	41,3065	0,0760	**0,0249**	0,4840	0,3586
CD8 TEMRA CD28	0,0337	0,0237	0,0121	0,0382	0,0621	**0,0007**	0,6029	**0,0256**	53,9868	40,1540	40,2346	40,0533	**0,0038**	**0,0066**	*0,0559*	0,9799
CD8 TEMRA CD56	0,0204	0,0218	0,0090	0,0377	0,5794	0,1926	0,6029	0,1783	24,2068	27,4496	30,2277	23,9769	0,2500	0,1028	0,8986	0,1947
CD8 TEMRA CD57	0,0266	0,0552	0,0147	0,1059	0,1333	0,7797	**0,0198**	**0,0330**	33,2839	45,7098	43,8182	48,0744	**0,0044**	**0,0195**	**0,0251**	0,5035
CD8 TEMRA CD127	0,0290	0,0486	0,0153	0,0903	0,8623	0,2508	0,0981	**0,0149**	50,5857	42,8957	40,6497	45,7032	**0,0278**	**0,0119**	0,3538	0,4592
CD8 TEMRA CX3CR1	0,0362	0,0396	0,0150	0,0704	0,2147	**0,0284**	0,7461	**0,0422**	43,2122	45,0755	45,5640	44,4242	0,7500	0,7462	0,8829	0,9078
CD8 TEMRA HLADR	0,0077	0,0151	0,0098	0,0218	0,9331	0,5358	0,3815	0,3688	12,1775	15,5350	18,4575	11,6384	0,1340	**0,0063**	0,5877	**0,0210**

Table S7. Absolute counts and percentages of TcRγδ cell subsets among study groups.

	ABSOLUTE NUMBERS								PERCENTAGES							
	mean				p-value				mean				p-value			
	HD	CAD	iCAD	ASCAD	HD vs CAD	HD vs iCAD	HD vs ASCAD	iCAD vs ASCAD	HD	CAD	iCAD	ASCAD	HD vs CAD	HD vs iCAD	HD vs ASCAD	iCAD vs ASCAD
TCRγδ CD25	0,0289	0,0013	0,0007	0,0021	**0,0000**	**0,0000**	**0,0000**	**0,0039**	12,1004	5,0222	2,4283	8,2647	**0,0003**	**0,0000**	0,3107	**0,0067**
TCRγδ CD127	0,0210	0,0215	0,0220	0,0209	0,3048	0,2438	0,6588	0,4786	55,4327	57,3943	57,4802	57,2870	0,3728	0,6890	0,7521	0,9775
TCRγδ CD56	0,0247	0,0165	0,0167	0,0161	**0,0010**	**0,0186**	**0,0024**	0,9875	32,1580	37,9548	34,5860	42,1659	0,1672	0,6160	0,0683	0,1972
TCRγδ CX3CR1	0,0183	0,0152	0,0167	0,0132	0,4132	0,7282	0,3001	0,4190	37,6266	35,2970	36,1582	34,1486	0,7018	0,7668	0,7513	0,7874
TCRγδ HLADR	0,0280	0,0087	0,0095	0,0076	**0,0000**	**0,0000**	**0,0000**	0,2979	22,8647	23,3545	24,4913	21,8387	0,8514	0,5567	0,7805	0,4690
TCRγδ CD27	0,0077	0,0126	0,0137	0,0111	**0,0000**	**0,0001**	**0,0018**	0,7176	45,9694	43,1563	43,3353	42,9327	0,5234	0,6405	0,6027	0,9551
TCRγδ CD28	0,0235	0,0136	0,0154	0,0112	**0,0010**	**0,0225**	**0,0018**	0,2758	49,2114	44,0948	47,9515	39,2741	0,2989	0,8457	0,0982	0,2581
TCRγδ CD57	0,0447	0,0163	0,0155	0,0173	**0,0000**	**0,0000**	**0,0000**	0,2901	28,7649	36,1716	30,2551	43,5671	0,2106	0,8721	**0,0149**	0,0620
TCRγδ CD28+CD57+	0,0015	0,0011	0,0008	0,0015	0,1136	**0,0091**	0,8196	**0,0199**	3,9248	4,5351	2,9199	6,5542	0,4165	0,6169	**0,0413**	**0,0101**
TCRγδ CD28+CD57-	0,0232	0,0125	0,0147	0,0097	**0,0004**	**0,0169**	**0,0004**	0,1681	45,2866	39,5597	45,0316	32,7199	0,2377	0,9683	**0,0254**	0,0958
TCRγδ CD28-CD57+	0,0168	0,0152	0,0148	0,0158	0,7397	0,4069	0,7138	0,3859	24,8402	31,6364	27,3352	37,0129	0,2319	0,9797	**0,0304**	0,1086
TCRγδ CD28-CD57-	0,0151	0,0110	0,0101	0,0120	**0,0322**	0,1811	**0,0274**	0,6258	25,9484	24,2687	24,7133	23,7131	0,7160	0,9257	0,6029	0,7866
TCRγδ CD28-CD57+CX3CR1+	0,0145	0,0076	0,0083	0,0067	0,2093	0,1926	0,5058	0,4582	19,3989	17,6440	15,2652	20,8159	0,8684	0,3725	0,4424	0,1222
TCRγδ CD28-CD56+CD57+CX3CR1+	0,0080	0,0054	0,0058	0,0049	0,3979	0,3693	0,6717	0,4993	10,9039	12,0443	10,3584	14,2921	0,6414	0,8441	0,2778	0,1905
TCRγδ CCR7	0,0006	0,0056	0,0002	0,0123	0,3155	0,5470	**0,0118**	**0,0101**	16,8120	17,4589	15,9072	19,3984	0,7925	0,8190	0,4612	0,2758
TCRγδ CD45RA	0,0102	0,0265	0,0293	0,0230	**0,0018**	**0,0032**	**0,0383**	0,2901	79,5748	67,5397	69,6787	64,8659	**0,0034**	**0,0219**	**0,0411**	0,5205
TCRγδ TN	0,0007	0,0014	0,0001	0,0031	0,7925	0,0796	**0,0111**	**0,0031**	16,3084	16,3760	15,4443	17,5405	0,9420	0,8588	0,7313	0,6711
TCRγδ TN CD25	0,0000	0,0001	0,0000	0,0002	0,1819	**0,0000**	**0,0100**	**0,0002**	12,1924	6,1780	3,6447	9,3445	**0,0019**	**0,0001**	0,3025	**0,0176**
TCRγδ TN CD27	0,0004	0,0005	0,0000	0,0011	0,0869	**0,0000**	**0,0034**	**0,0000**	56,3141	42,6519	40,4125	45,4511	**0,0157**	**0,0172**	0,1334	0,5695
TCRγδ TN CD28	0,0003	0,0004	0,0000	0,0009	0,4295	**0,0004**	**0,0087**	**0,0010**	46,5998	35,7062	37,0458	34,0317	**0,0132**	0,1339	*0,0551*	0,7124
TCRγδ TN CD56	0,0001	0,0007	0,0000	0,0015	0,5948	0,0607	**0,0013**	**0,0002**	23,4007	37,4364	33,7081	42,0967	**0,0027**	0,1224	**0,0004**	0,2183
TCRγδ TN CD57	0,0001	0,0009	0,0001	0,0019	**0,0477**	0,7282	**0,0000**	**0,0003**	23,6433	42,5555	37,2583	49,1771	**0,0001**	**0,0310**	**0,0000**	0,0940
TCRγδ TN CD127	0,0005	0,0010	0,0002	0,0019	0,1220	0,5584	**0,0004**	**0,0002**	75,0721	74,8639	77,6953	71,3248	0,9282	0,7131	0,7966	0,2845
TCRγδ TN CX3CR1	0,0002	0,0007	0,0001	0,0014	0,8799	*0,0561*	**0,0118**	**0,0013**	30,4915	30,6775	29,6698	32,0211	0,3283	0,1811	0,8106	0,5869

TCRγδ TN HLADR	0,0003	0,0008	0,0000	0,0019	0,9331	0,0683	**0,0221**	**0,0060**	45,6261	47,4139	43,2869	52,9166	0,8148	0,6289	0,3183	0,0861
TCRγδ TM	0,0017	0,0071	0,0005	0,0153	0,3912	0,1496	**0,0010**	**0,0003**	83,6916	83,6240	84,5557	82,4595	0,9420	0,8588	0,7313	0,6711
TCRγδ TM CD25	0,0010	0,0006	0,0000	0,0014	0,0946	**0,0000**	*0,0501*	**0,0000**	11,5853	4,8573	2,3210	8,0276	**0,0067**	**0,0001**	0,7313	**0,0083**
TCRγδ TM CD27	0,0010	0,0036	0,0000	0,0082	0,1220	**0,0000**	**0,0221**	**0,0003**	44,8403	44,9386	44,2579	45,7894	0,9830	0,9171	0,8833	0,8390
TCRγδ TM CD28	0,0019	0,0034	0,0004	0,0071	0,9331	0,0657	**0,0478**	**0,0074**	50,2762	46,5273	49,9503	42,2486	0,4654	0,9606	0,2061	0,3269
TCRγδ TM CD56	0,0012	0,0032	0,0003	0,0068	0,5416	0,1868	**0,0076**	**0,0022**	32,7852	36,9136	34,2399	40,2558	0,3048	0,7155	0,1700	0,3255
TCRγδ TM CD57	0,0011	0,0031	0,0007	0,0062	0,1869	0,2958	**0,0002**	**0,0002**	29,3188	34,3383	29,1532	40,8197	0,4101	0,7282	0,0605	0,0949
TCRγδ TM CD127	0,0015	0,0040	0,0006	0,0083	0,3048	0,1648	**0,0004**	**0,0001**	51,8572	53,5681	53,5864	53,5451	0,4768	0,7479	0,7810	0,9955
TCRγδ TM CX3CR1	0,0017	0,0027	0,0006	0,0054	0,5948	0,1985	**0,0132**	**0,0019**	37,4875	35,5613	37,1493	33,4440	0,8594	0,9123	0,8621	0,6227
TCRγδ TM HLADR	0,0005	0,0014	0,0002	0,0028	0,4495	0,3818	**0,0167**	**0,0060**	18,9733	19,0020	22,0840	14,8927	0,9898	0,1809	0,1051	**0,0085**
TCRγδ TCM	0,0002	0,0002	0,0000	0,0004	0,1708	0,8507	**0,0289**	**0,0355**	0,8095	1,0829	0,4629	1,8579	**0,0171**	0,1041	**0,0212**	0,1148
TCRγδ TCM CD25	0,0000	0,0000	0,0000	0,0000	0,9173	0,6502	0,4686	0,5248	26,8837	19,8035	26,0963	11,9376	0,4996	0,8615	0,1589	0,1333
TCRγδ TCM CD27	0,0001	0,0001	0,0000	0,0003	0,4787	0,3989	**0,0255**	**0,0253**	64,3518	57,8280	64,7926	49,1222	0,1934	0,5419	0,1155	0,1336
TCRγδ TCM CD28	0,0002	0,0001	0,0000	0,0003	0,5727	0,9250	0,2652	0,3226	78,1766	69,6157	84,1486	51,4495	0,1126	0,8465	**0,0028**	**0,0038**
TCRγδ TCM CD56	0,0000	0,0001	0,0000	0,0002	0,1940	0,9432	**0,0273**	*0,0599*	16,4940	24,3942	20,2458	29,5796	0,1939	0,5270	0,1195	0,3723
TCRγδ TCM CD57	0,0000	0,0000	0,0000	0,0001	0,1477	0,5136	**0,0022**	**0,0027**	3,5387	8,2658	4,3152	13,2039	0,2461	0,8469	**0,0281**	**0,0424**
TCRγδ TCM CD127	0,0001	0,0001	0,0000	0,0003	0,0960	0,5625	**0,0006**	**0,0007**	61,3666	68,7869	79,6865	55,1623	0,3615	*0,0507*	0,5235	**0,0157**
TCRγδ TCM CX3CR1	0,0000	0,0000	0,0000	0,0001	**0,0259**	0,3977	**0,0029**	**0,0215**	9,0581	17,2211	17,0593	17,4370	0,0950	0,4318	**0,0390**	0,3056
TCRγδ TCM HLADR	0,0000	0,0000	0,0000	0,0001	**0,0271**	0,3985	**0,0033**	**0,0215**	15,4814	41,0409	54,2164	23,4737	**0,0001**	**0,0000**	0,1505	**0,0012**
TCRγδ TEM	0,0003	0,0025	0,0000	0,0056	**0,0015**	0,0659	**0,0005**	**0,0048**	19,6052	31,3774	29,8584	33,2762	**0,0030**	**0,0161**	**0,0476**	0,6370
TCRγδ TEM CD25	0,0001	0,0007	0,0000	0,0015	0,8535	0,0957	**0,0221**	**0,0044**	15,6127	6,4064	4,7694	8,4527	**0,0094**	**0,0034**	0,2253	0,1187
TCRγδ TEM CD27	0,0002	0,0017	0,0000	0,0039	0,6828	0,1596	**0,0167**	**0,0123**	63,0464	61,3538	61,5746	61,0779	0,4505	0,6832	0,7447	0,9321
TCRγδ TEM CD28	0,0005	0,0019	0,0001	0,0042	0,1652	0,9527	**0,0104**	**0,0083**	80,8844	75,6871	81,6645	68,2152	0,1765	0,8059	**0,0074**	**0,0196**
TCRγδ TEM CD56	0,0001	0,0016	0,0000	0,0035	0,0880	0,9797	**0,0024**	**0,0031**	20,8737	27,8063	21,7840	35,3343	**0,0382**	0,5470	**0,0028**	**0,0130**
TCRγδ TEM CD57	0,0000	0,0010	0,0000	0,0023	*0,0555*	0,8912	**0,0004**	**0,0010**	10,9748	12,2619	7,8649	17,7581	0,2437	0,5552	**0,0060**	**0,0005**
TCRγδ TEM CD127	0,0002	0,0015	0,0000	0,0033	0,0922	0,8042	**0,0011**	**0,0017**	50,0982	56,4470	56,9014	55,8791	0,1710	0,2493	0,3441	0,8896
TCRγδ TEM CX3CR1	0,0001	0,0011	0,0000	0,0025	0,1341	0,6815	**0,0314**	**0,0400**	18,2676	22,8793	24,5282	20,8182	0,4109	0,3925	0,6554	0,7623
TCRγδ TEM HLADR	0,0000	0,0005	0,0000	0,0010	0,1728	0,7323	**0,0462**	0,0625	21,8392	19,7403	25,6116	11,9118	0,7000	0,0890	**0,0058**	**0,0044**
TCRγδ TEMRA	0,0010	0,0034	0,0001	0,0075	0,3492	0,4813	**0,0118**	**0,0067**	63,2769	51,1637	54,2344	47,3254	**0,0054**	**0,0356**	**0,0238**	0,3136

TCRγδ TEMRA CD25	0,0005	0,0008	0,0000	0,0017	**0,0159**	**0,0000**	0,1975	**0,0000**	10,6367	4,4319	1,3061	8,3391	**0,0025**	**0,0000**	0,7361	**0,0005**
TCRγδ TEMRA CD27	0,0005	0,0014	0,0000	0,0032	**0,0341**	**0,0000**	**0,0140**	**0,0000**	40,1577	39,8807	38,6404	41,4311	0,9517	0,8010	0,8196	0,6954
TCRγδ TEMRA CD28	0,0008	0,0010	0,0001	0,0022	0,2363	**0,0008**	0,0941	**0,0060**	40,8478	34,1829	36,1188	31,7630	0,1700	0,4602	0,1089	0,5424
TCRγδ TEMRA CD56	0,0006	0,0016	0,0001	0,0035	0,8185	**0,0196**	**0,0221**	**0,0019**	36,0902	40,2602	39,2588	41,5119	0,3506	0,5502	0,3221	0,7023
TCRγδ TEMRA CD57	0,0009	0,0023	0,0007	0,0043	0,3728	0,2370	**0,0024**	**0,0013**	34,3805	43,4645	36,3944	52,3020	0,1146	0,8855	**0,0047**	0,0887
TCRγδ TEMRA CD127	0,0010	0,0019	0,0004	0,0037	0,7497	0,0683	**0,0063**	**0,0008**	52,6506	52,5309	53,1512	51,7555	0,9791	0,9269	0,8904	0,8512
TCRγδ TEMRA CX3CR1	0,0012	0,0016	0,0004	0,0032	0,9598	0,0657	**0,0413**	**0,0031**	43,2066	40,7316	43,0843	37,7908	0,7412	0,9797	0,5893	0,4749
TCRγδ TEMRA HLADR	0,0003	0,0009	0,0001	0,0018	0,9331	0,0738	*0,0551*	**0,0163**	18,8834	18,2154	19,9863	15,8543	0,7851	0,6717	0,3830	0,2411

Table S8. CMV contingency table

	CMV+ (n)	CMV- (n)
iCAD	20	0
ASCAD	14	2
Total	34	2

Fisher's exact test

The two-tailed P value equals 0.1905
The association between rows (groups) and columns (outcomes)
is considered to be not statistically significant.
